# Economists: moral realists or real moralists? Comment on Fourcade and Brunetti

**DOI:** 10.1186/s41937-017-0018-3

**Published:** 2018-01-25

**Authors:** Monika Bütler

**Affiliations:** University of St. Gallen, SEW-HSG, Varnbüelstrasse, 14, 9000 St. Gallen, Switzerland

A comparative sociologist who has spent her international career in academia—Marion Fourcade—and a practically minded academic economist with an extended stint in the Swiss government—Aymo Brunetti—offer their views on the role of economists in politics and society. Although Fourcade and Brunetti differ a lot in terms of experience and interest, their insightful and nuanced assessments of economics today are often remarkably similar.

Marion Fourcade addresses economists with the Japanese attitude of a 30-degree bow. She modestly calls her views “from below” and she refrains from throwing the gauntlet to the economics profession, as many representatives of social sciences have done in the past. That does not mean she turns a blind eye on some weaknesses of our field. Yet, she portraits problematic issues as interesting aspects of economics. Her text is a pleasure to read because it is an invitation to think about our field, rather than a challenge to a duel.

More hands on, but not less interesting, Aymo Brunetti features another tension economists face: not between academic fields, but rather within the economics profession between academically and more policy-oriented scholars. Interestingly, both non-economists and economic practitioners often feel to be looked down upon by academic economists.

Marion Fourcade’s label the “Veiled Moralists” is certainly appropriate for many, if not all, representatives of our profession. Gunnar Myrdal made a big effort to distinguish positive and normative economics and to warn his fellow economists of the gateways through which hidden value judgements enter into and contaminate our analysis. However, the dichotomy positive-normative is too narrow. Thomas Kuhn, in his analysis of “scientific revolutions”, found that, before drawing positive or normative conclusions, scientists need a way of looking at the world, a pair of glasses he called “paradigms”. A typical example would be Hayek’s “decentralized information” view, as Marion Fourcade calls it.

A paradigm is not a value statement. Yet it can hardly ever be normatively or politically “clean”. The decentralized information paradigm, e.g. is quite conducive to pro-market attitudes: the market is viewed as a “regime of truth-telling”, a term coined by French historian Michel Foucault. “Moralism”, therefore, must be hidden on the very DNA of economics (and—of course—other social disciplines).

Economists are a fortunate crowd, as is clear from Aymo Brunetti’s contribution: They can rely (not without limits) on the power of the price mechanism, following Adam Smith’s “Invisible Hand”. None of the other social sciences seems to benefit from a similar source of rather robust first approximations. Of course, as any paradigm, it is open for ideological contamination. Yet, few would reject the view that the price mechanism can help to tackle many of today's challenges like climate change, congestion and demographic change.

Interestingly, Marion Fourcade does not emphasize the price mechanism itself, but rather money as the unit of account, which turns qualities into quantities. However, as she rightly points out, money comes with its “own moral bag”. Her analysis is reminiscent of Aristotle’s difference between economics and chrematistics. For Aristotle, money must only be a medium of exchange and measure of value (economics), while the accumulation of money as the ultimate goal is an unnatural activity that dehumanizes those who practice it (chrematistics). Although economists are eager to point out that (individual) utility must be the goal of any policy intervention, rather than money, the perception by the public is a different one. The “money is everything” view seems to be justified by the high rate of return of a degree in economics. (The average masks a large heterogeneity among economists, however: While some go for gold, others can be lucky to get a glass of water and have their travel expenses reimbursed for their advice—which makes them closer to sociologists, I guess).

Are economists “fortune tellers” as Marion Fourcade suggests? The failure of most economists to predict the two big cases of the Great Depression and the recent Financial Crisis suggests that their fortune telling abilities are limited. More importantly, crisis and scandals around some famous representatives of the field have led to a loss of faith in economics itself. But, as Aymo Brunetti adds, (unforeseen) crises do not mean that the science of economics is inherently unreliable. There are many examples where economic reasoning has indeed led to accurate predictions and useful policy measures. Others—such as some crisis in the Euro Area—occurred because economists ignored what they knew, or because they were ignored by politicians. Marion Fourcade comes to the most powerful (and flattering) aid of economists: a counterfactual usually does not exist (especially in macroeconomics). We cannot know what would have happened if economists had not offered their advice. As comforting the support from another discipline may be, it will not spare economists from blame in the future for simple reasons: Not only do failures stand out more than the successes, the “dismal science” (Thomas Carlyle) is often also the bearer of bad news.

The “denegation of politics”, as Marion Fourcade calls it, and Aymo Brunetti illustrates with examples, is a denegation of our very identity of economists. There is no immaculate conception of economic theories. Although economists are eager to distance themselves from politics, they are still considered as the most influential social science. I wonder whether this perception reflects the real world. Politicians rarely use economic knowledge to make decisions or set new laws. Apart from central banks, most bodies who decide the economic fate of a country (or a group of countries) do not include trained economists (and rarely rely on sound economic advice, one might add). The strive to be perceived as apolitical has the consequence that economic knowledge is overlooked and that economic advice is often left to pseudo economist. As someone often venturing out into the “real world”, I can also add that, very often, giving economic advice can be frustrating. Only in rare cases, the sought after advice is based on one’s own cherished academic research. In the majority of situations, basic economic principles do the trick, or even more daunting, advice is tantamount to the detection of mistakes in economic reasoning. Yet, the authority of an academic economist still counts.

Is economics really influential, one could ask. Many observers from the inside and outside of the field would confine the impact of academic economic research to the ivory tower. Indeed, the choice of topics in academic papers seems to reflect personal interests, data availability and cool identification strategies more than the relevance of real-world economic problems. To some degree, such a phenomenon is unavoidable in any basic research. But the choice of research topics and biases in views and fields also derive from a pretty undiversified faculty dominated by US and European scholars with a low share of women.

Communication is key, as Aymo Brunetti forcefully stresses. One way economists have seen their public impact rise is through blogs and commentaries. But the communication to the public being a public good, many platforms have seen declining the number of contributions by the best scholars. Even modern communication tools cannot resolve the basic trade-off between time devoted to academic research and other activities.

On a brighter side, academic research has seen a rise in interest, perhaps not from politics, but from other fields. A recent analysis of citations to and from other disciplines (Angrist et al. 2017) demonstrates that economics papers increasingly cite non-economic research, and other disciplines cite economists more often too. The authors attribute their findings to a rising quantity and quality of empirical research in our field which has increased the relevance of the field to non-economists. Yet, a very welcome compilation of materials to illustrate concepts in economics by the American Economic Association, tellingly labeled “real-world economics”, reveals a dominance of microeconomic applications and a striking underrepresentation of questions in macroeconomics and public finance.

What to do? Marion Fourcade, in her perspective “from below”, does not propose fields for the cooperation of economists and sociologists and other social sciences. She may have good reason. The call for “interdisciplinary” research is often a label helpful in the fight for funding. Yet, it is difficult to wear two pairs of glasses (paradigms) at the same time. Not even the behavioral economists did so. They found some results psychologists already knew or would have predicted, but they did so by way of controlled and repeatable experiments inspired by micro-economic thinking.

We just should learn more from each other, from other disciplines and from practical economists closer to the real world. Instead of taking a “view from below”, we should take a look from the side—from all sides, to be sure.
